# The effect of Sailuotong (SLT) on neurocognitive and cardiovascular function in healthy adults: a randomised, double-blind, placebo controlled crossover pilot trial

**DOI:** 10.1186/s12906-016-0989-0

**Published:** 2016-01-13

**Authors:** Genevieve Z. Steiner, Alan Yeung, Jian-Xun Liu, David A. Camfield, Frances M. de Blasio, Andrew Pipingas, Andrew B. Scholey, Con Stough, Dennis H. Chang

**Affiliations:** 1 National Institute of Complementary Medicine, and School of Science and Health, Western Sydney University, Penrith, NSW 2751 Australia; 2 Centre for Psychophysics, Psychophysiology, and Psychopharmacology; Brain & Behaviour Research Institute; and School of Psychology, University of Wollongong, Wollongong, NSW 2522 Australia; 3 Xiyuan Hospital, China Academy of Chinese Medical Sciences, Beijing, 100091 China; 4 Centre for Human Psychopharmacology, Swinburne University of Technology, Hawthorn, VIC 3122 Australia; 5 Illawarra Health & Medical Research Institute, University of Wollongong, Wollongong, NSW 2522 Australia

**Keywords:** Sailuotong (SLT), Chinese Herbal Medicine (CHM), EEG, Event-related potentials (ERPs), P3(00), Blood pressure, Heartrate (HR), Neurocognition, Age related cognitive decline

## Abstract

**Background:**

Sailuotong (SLT) is a standardised herbal medicine formula consisting of *Panax ginseng*, *Ginkgo biloba*, and *Crocus sativus*, and has been designed to enhance cognitive and cardiovascular function.

**Methods:**

Using a randomised, double-blind, placebo controlled crossover design, this pilot study assessed the effect of treatment for 1 week with SLT and placebo (1 week washout period) on neurocognitive and cardiovascular function in healthy adults. Sixteen adults completed a computerised neuropsychological test battery (Compass), and had their electroencephalographic (EEG) activity and cardiovascular system function assessed. Primary outcome measures were cognitive test scores and oddball task event-related potential (ERP) component amplitudes. Secondary outcome measures were resting EEG spectral band amplitudes, and cardiovascular parameters.

**Results:**

Treatment with SLT, compared to placebo, resulted in small improvements in working memory, a slight increase in auditory target (*cf.* nontarget) P3a amplitude, and a decrease in auditory N1 target (*cf.* nontarget) amplitude. There was no effect of SLT on EEG amplitude in delta, theta, alpha, or beta bands in both eyes open and eyes closed resting conditions, or on aortic and peripheral pulse pressure, and resting heartrate.

**Conclusions:**

Findings suggest that SLT has the potential to improve working memory performance in healthy adults; a larger sample size is needed to confirm this.

**Trial registration:**

Australia New Zealand Clinical Trials Registry Trial Registration Id: ACTRN12610000947000.

## Background

Most individuals experience a normal age-related decline in a range of cognitive abilities including a decrease in information processing speed, and learning and memory abilities [1]. For instance, cross-sectional studies have demonstrated that working memory ability decreases across most of the adult lifespan, with sharper declines after 70 years of age [2]. Management of age-related changes in cognition can be challenging as symptoms can closely overlap with early stages of dementia, such as Alzheimer’s disease, which has a different pathophysiology; for example, the presence of neurofibrillary tangles and beta-amyloid plaques [3].

The ageing process can also be associated with neurological pathology [4], particularly at a cellular level. For instance, a loss of synaptic density can contribute to a reduction in regional brain volume and cortical thickness [5–7], with some regions that are responsible for advanced cognition (e.g., working memory), including the prefrontal cortex, showing a steady decline after the age of 20 [1]. Other factors linked to age-related cognitive decline (and possibly the pathogenesis of dementia) include changes in brain metabolite concentrations, inflammation, oxidative stress, brain vascular integrity, and poor circulation, which may be associated with a number of disease-related and lifestyle-related risk factors such as diabetes, cardiovascular disease, obesity, and excessive alcohol consumption [8–12].

In order to minimise the functional impairment associated with the ageing process, particularly slowing, stabilising, or (possibly) reversing cognitive decline, there is a demand for appropriate pharmacological interventions. Currently, there are few pharmaceutical options available for otherwise healthy individuals, and these are usually prescribed under the assumption of a pathophysiological relationship between cognitive decline and dementia [13]. For example, acetylcholinesterase (AChE) inhibitors (e.g., donepezil) act on the enzyme which breaks down acetylcholine, increasing its availability in the synapses [14]; and N-methyl-D-asparate (NMDA) glutamate receptor antagonists (e.g., memantine) normalise the glutamatergic system by blocking NMDA glutamate receptors [15]. There is little evidence to suggest that these pharmacological approaches can effectively treat forms of cognitive decline other than dementia, including mild cognitive impairment [16–18] which is often considered the prodromal phase for Alzheimer’s disease [13]. AChE inhibitors are also poorly tolerated, with adverse side effects related to cholinergic hyperactivity including bradycardia, hypotension, gastrointestinal disturbances, vomiting, and bronchoconstriction [19], making their implementation ethically unsound in populations that are otherwise unimpaired [20]. Further, the global changes associated with age-related cognitive decline mentioned above indicate the need for multi-target [21], rather than single-target treatments.

Where standard pharmaceuticals often utilise a single-target approach (e.g., AChE inhibitors), complementary medicine treatments, such as Chinese Herbal Medicine (CHM), apply a multisystem approach in which combination multi-target therapies are used to treat disease [22]. Complex herbal formulations used in CHM can also have a synergistic effect, where additional extractions increase the efficacy of the formula as a whole [23–27]. Given this approach, CHM may be more suitable in the treatment of age-related cognitive decline, and is well-suited for chronic interventions due to accumulative effects on multiple systems over a period of time. Further, early research suggests that some herbal formulations can enhance memory function and increase longevity [28].

Sailuotong (SLT) is a new standardised three-herb formula that combines specific doses of the key bioactive constituents from the concentrated extracts of *Panax ginseng* (ginsenosides), *Ginkgo biloba* (flavone-glycosides), and *Crocus sativus* (crocins), and has been developed to enhance cognitive and cardiovascular function through multiple pathways of action based on a comprehensive review of the CHM and medical literature. The three herbal constituents have each demonstrated neuroprotective, antioxidant, and anti-hypertensive properties [29–32], and preliminary research suggests the possibility of an enhanced therapeutic effect due to synergism [23–25].

Preclinical pharmacokinetic, pharmacodynamic, and toxicity studies have been conducted on these three herbs separately, and in combination [29–38]. Treatment with SLT in various experimental ischemia and amnesia models in rodents resulted in improved learning and memory function, antioxidant capacity, and pathogenic biochemical blood and brain tissue parameters. For example, over an eight week period, SLT decreased the latency for finding the platform in a Morris Water Maze in rats with chronic cerebral hypoperfusion model induced by bilateral common carotid artery ligation [35]. Brain tissue cholinesterase decreased and acetylcholine levels increased, and importantly, superoxide dismutase (an enzyme that acts as an antioxidant) also increased. In a similar investigation using an amyloid beta-protein induced dementia model in mice [36], brain tissue acetylcholine increased by 18.56 and 19.97 %, respectively, when treated for 30 days with low (15.5 mg/kg) and high (31.0 mg/kg) doses of SLT. Another study using a PDAPP^v7171^ transgenic dementia model in mice showed that treatment for 12 weeks with both low (31 mg/kg) and high (62 mg/kg) doses of SLT increased brain tissue acetylcholine levels [31]; brain tissue serotonin levels decreased in the high dosage group only.

A range of cardiovascular benefits of SLT have also been demonstrated [29, 30, 33]. Acute treatment over 24 h decreased the areas of focal cerebral ischemia/reperfusion injury in mice, and increased cerebral blood flow was also observed 60–180 min after administration (10 mg/kg) [33]. Rats also showed a decrease in platelet aggregation rate and whole blood viscosity after treatment with low (8 mg/kg) and high (16 mg/kg) doses of SLT for 7 days.

In humans, a 16 week pilot study of SLT was conducted on individuals with probable or possible vascular dementia [38]. Participants treated with SLT showed a significant improvement in Alzheimer’s Disease Assessment Scale cognitive subscale (ADAS-cog) scores compared to placebo. Participants also reported significant improvements in quality of life (Short Form Health Survey; SF-36) related to emotional and physical role functioning, mental health, and social functioning. A subset of participants also underwent single photon emission computed tomography (SPECT) scan, which demonstrated that those treated with SLT, compared to placebo, showed increased blood flow in the inferior frontal and anterior temporal lobes, which was greater in the left hemisphere. This is a particularly promising finding, as those regions are associated with memory function, and auditory and speech processing.

Due to the lack of treatment options available for age-related cognitive decline, and SLT’s potential to improve cognitive and cardiovascular function, a trial of SLT on healthy individuals is warranted. The current pilot study employed a randomised, double-blind, placebo controlled crossover design, and aimed to provide preliminary data on the possible cognitive and cardiovascular benefits of SLT in healthy adults. Neurocognitive function was assessed using a computerised cognitive test battery, oddball task event-related potential (ERP) component amplitudes, and resting EEG spectral band amplitudes. Cardiovascular system function measures were central and peripheral pulse pressure, and resting heartrate (HR). It was hypothesised that treatment for 1 week with SLT would improve neurocognitive and cardiovascular function compared to placebo.

## Methods

### Inclusion criteria

Sixteen adults were recruited from students, staff, and their families at Western Sydney University through advertisements on campus. All provided informed consent and were advised that they were free to withdraw at any time without penalty. Participants were excluded if they were pregnant, obese (to reduce the chance of recruiting participants with associated chronic diseases), had a history of allergies, serious gastrointestinal disorders, asthma or serious pulmonary disorders, diabetes, or any significant abnormality on laboratory tests including full blood count, liver, and renal function. Any participants taking anti-coagulant or cognitive enhancing medication/substances, including over the counter *Ginkgo biloba* or *Panax ginseng* capsules, were also excluded. All participants were non-smokers and showed no signs of neurological impairment (mini mental state exam; MMSE > 28).

### Treatments

*SLT treatment*: SLT formula is standardised by the bioactive components of the three herbal extracts from *Panax ginseng*, *Ginkgo biloba,* and *Crocus sativa*. A range of in vivo pharmacological studies have been undertaken to test the bioactivity and determine the optimal ratio of the three individual herbal extracts. Each SLT capsule contains a 60 mg standardised mixture of 27.27 mg ginsenosides from *Panax ginseng*, 27.27 mg total ginkgo flavone-glycosides from *Ginkgo biloba*, and 5.46 mg crocins from *Crocus sativa*. The SLT preparations were manufactured in a Good Manufacturing Practice certified facility.

*Placebo*: Two capsules per day, containing an inert substance matched for the colour, taste, and smell of SLT.

### Design

This pilot study employed a randomised, placebo controlled, double-blind crossover design. Participants were required to orally ingest two capsules of either SLT or a placebo every day for one week. This was followed by a one week washout period and the subsequent counterbalanced treatment week. This washout period length was determined by preclinical pharmacokinetic studies [33]. The active herbal formulation and placebo were prepared in an extract capsule form in accordance with Australian Goods Manufacturing Practices. Neurocognitive and cardiovascular function were tested before and after each of the interventions. Primary outcome measures included neurocognitive function test scores, and ERP component amplitudes. Secondary outcome measures were EEG spectral band amplitudes, aortic and peripheral pulse pressure, and resting HR. The procedure was approved by Western Sydney University's Human Research Ethics Committee (Ethics Approval: H8253), and the trial was registered with the Australian New Zealand Clinical Trials Registry (Trial Id: ACTRN12610000947000).

### Materials and apparatus

#### Cognitive test battery

Cognitive function was tested using the Computerised Mental Performance Assessment System (Compass) neuropsychological test battery, which measures a range of cognitive abilities including attention, episodic memory, and working memory. These cognitive domains were assessed using 12 Compass tasks: Immediate Word Recall, Delayed Word Recall, Word Recognition, Simple Reaction Time, Choice Reaction Time, Numeric Working Memory, Alphabetic Working Memory, Corsi Block Span, N-Back Working Memory, Picture Recognition, Face Recognition, Serial Subtraction, and Rapid Visual Information Processing (RVIP). Parallel versions of each task were used for the repeated testing sessions.

Mental fatigue and Mood were self-reported on a Visual Analogue Scale (VAS) using a 100 mm horizontal line displayed on a computer monitor, with each endpoint marking the two extremes of mental fatigue or mood; participants responded using a computer mouse cursor. Three dimensions of mood were assessed (alertness, calmness, and contentment) with 16 separate Bond-Lader VAS items [39].

#### Electroencephalograph (EEG)

EEG data were recorded continuously from 18 scalp sites (F1, F3, Fz, F2, F4, C1, C3, Cz, C2, C4, P1, P3, Pz, P2, P4, O1, Oz, O2) with a 64 channel electrode cap using sintered Ag/AgCl electrodes. The nose was used as a reference and the cap was grounded by an electrode located midway between AF3 and AF4. Data were acquired 0–200 Hz using a Neuroscan Synamps 2 digital signal-processing system and Neuroscan 4.3.1 Acquire software. The display and stimulus markers were controlled by a separate stimulus computer (Dell Optiplex 760 with a 22 " LG Flatron W2253TQ screen) using Compumedics Stim2 (4.0.09302005) software.

Electro-oculogram (EOG) was recorded using sintered Ag/AgCl electrodes placed 2 cm above and below the left eye for vertical movements, and on the outer canthus of each eye for horizontal movements. Impedance was less than 10 kΩ for cap, EOG, and reference electrodes. Scalp and EOG potentials were amplified with a gain of 2816 and digitised at 1000 Hz.

EEG was recorded during two resting conditions (5 min eyes open and 5 min eyes closed) followed by two oddball tasks (visual then auditory). For the visual oddball task, stimuli were white 37 × 43 mm letters (target: X; nontarget: O) presented for 500 ms on a black background. For the auditory task, acoustic stimuli were delivered binaurally through foam stereo eartips (ER3-15A) using the Stim Audio System (P/N 1105), and consisted of 1000 Hz (target) and 500 Hz (nontarget) 80 dB tones (500 ms duration, 10 ms rise/fall time). For each of these tasks, a randomised stimulus sequence consisting of 200 trials was presented (target *p* = .2, nontarget *p* = .8) with an ISI of 1.5 s in a single block that was approximately 5 min in duration. Targets were responded to with a button press using the Stim System Switch Response Pad (P/N 1141).

#### Cardiovascular measures

Aortic and peripheral measures of pulse pressure, and resting HR were assessed using an electronic sphygmomanometer and tonometer (AtCor Medical SphygmoCor CVMS v8.2); the mean across three consecutive measurements of each index was computed. To ensure reliability, only measurements equal to or greater than an Operator Index of 80 were used. The Operator Index is a measure of quality control derived from the mean and variability in pulse strength, diastolic variation, and systolic shape deviation. The score is out of a possible 100, and an Index over 80 is considered acceptable.

### Procedure

Participant eligibility was confirmed during a brief telephone interview. Then, during a screening session, participants provided informed consent and completed the MMSE before a medically trained professional conducted a medical questionnaire, physical examination, and provided participants with a pathology request form so that blood samples could be taken and analysed at a Douglas Hanley Moir pathology laboratory. Blood sampling was only deemed necessary for participants aged 50–75; pathology examined were full blood count, and liver and kidney function. If no abnormalities were discovered participants completed a training session on the Compass test battery, and were randomly allocated to a treatment group via a computer randomisation package. The randomisation was conducted by the research program coordinator externally to the research team and the assignments were blinded to all investigators and assessors as well as to the trial participants. All data were collect at the National Institute of Complementary Medicine Clinical Laboratory, Western Sydney University during 2012–2013.

At the beginning of each of the one-week intervention cycles, participants attended a baseline session where they were asked a series of questions about any illnesses or adverse events, and whether they had started, stopped, or changed any medications. Then, whilst sitting comfortably, participants had their cardiovascular measurements taken, completed the Compass testing battery, were fitted with EEG recording apparatus, and had their EEG activity recorded. Each of these baseline sessions ran for approximately 2.5 h. Efforts were made to ensure that each timepoint for each participant was at the same time of day. Participants were dispensed eighteen capsules containing either the SLT formulation or placebo (depending on randomisation) and were instructed to take two capsules per day for one week (morning and night with food), and to return any unused capsules at the post-treatment session. Four extra capsules were provided in the event that participants were unable to attend the next scheduled appointment. Halfway through the treatment cycle, a brief telephone follow-up was conducted to ensure participant safety in regards to adverse reactions and illness, and to confirm the time and date of the next testing session. The post-treatment sessions were identical to the baseline, with the exception that participants returned any excess capsules and also completed a short survey to determine the successfulness of the blinding procedure.

After the initial intervention cycle, there was a seven day washout period to allow for the elimination of any residual herbal compound from the body. Following this, participants began a second week of treatment in which they received the counterbalanced treatment condition (SLT or placebo), and again were tested pre- and post-intervention cycle.

#### EEG data extraction and quantification

Using Neuroscan Edit software (Compumedics, Version 4.3.1), the EEG data were EOG artefact [40] and DC-offset corrected, and low-pass filtered at 30 Hz (24 dB/Octave). Any additional artefacts exceeding ± 100 μV were excluded. Oddball task data were epoched from 200 ms pre- to 1400 ms post-stimulus and baseline corrected using the pre-stimulus period. Trials containing omission (misses) and commission (false alarms) errors were excluded. For each subject and each modality, averages were computed for each of the four treatment sessions and the two stimulus conditions. Averaged half-sampled data (epoched −100 to 600 ms: 350 datapoints) from 18 scalp locations were submitted to two separate temporal Principal Components Analyses (PCAs; one for each modality) using the ERP PCA toolkit (v. 2.23 [32]) in MATLAB (The Mathsworks; Version 8.0.0.783, R2012b). In each of the PCAs, factors for all conditions were quantified simultaneously (2304 observations: 16 participants × 2 stimulus conditions × 4 testing sessions × 18 electrodes). The PCAs used the unstandardised covariance matrix with Kaiser normalisation, and all 350 unrestricted factors underwent Varimax rotation [41]. PCA factors were identified as ERP components based on their latency, topography, and polarity of their conspicuous maximum loading. The peak amplitude of each identified component was output and entered into subsequent statistical analyses.

The 5 min of artefact-corrected data from each of the resting conditions were epoched to 2000 ms and then quantified in MATLAB and EEGLAB (Version 9.0.8.6b [42]). For each subject and each condition, spectral amplitudes were computed using discrete Fast Fourier Transforms with a 10 % Hanning window for each of the four treatment sessions. Frequency data from 0 to 30 Hz were extracted with a resolution of 0.5 Hz, before averaging across epochs. The spectral band amplitudes were calculated by summing the activity across the frequency bins for delta 0.5-3.5 Hz, theta 4.0-7.5 Hz, alpha 8.0-13.0 Hz, and beta 13.5-29.5 Hz.

### Statistical analyses

As this was a pilot trial, no formal sample size calculation was made. Sample size was approximated based on a trial [65] which employed two of the three SLT constituents as a combined intervention using a similar cross-over design. A post-hoc sample size calculation showed that in order to detect a difference of *d* = 1.00 at 80 % power, two-tailed, α = .05, 17 participants were required, suggesting that the sample size used here, although small, was sufficient to detect a meaningful change in the primary outcome measure.

#### Cognitive and cardiovascular measures

Relevant outcome measures were recorded automatically by the Compass and SphygmoCor software. Separate repeated measures analyses of variance (ANOVAs) with *Treatment* (SLT vs. placebo) and *Time* (baseline vs. post-treatment) as within-subject factors was used to compare the effects of SLT with placebo for each of the Compass and cardiovascular measures. As this was a pilot study, results approaching significance (*p* < .10) are reported as they may potentially reflect the existence of real effects.

#### EEG and ERPs

Expected EEG band and ERP component topographies were defined by selecting a cluster of electrodes surrounding the site of maximal amplitude. Using a mean across a region defined by multiple sites, rather than a single electrode, reduces the impact of chance variance at a single site [43].

Separate repeated-measures MANOVAs then assessed each band and component’s amplitude for the effects of *Condition* (EEG: eyes open vs. eyes closed; ERPs: target vs. nontarget), *Treatment* (SLT vs. placebo) and *Time* (baseline vs. post-treatment). Cohen’s *d* effect sizes are reported. The violations of sphericity assumptions associated with repeated-measures analyses do not affect single degree of freedom contrasts, so Greenhouse-Geisser-type correction was not necessary [44]. All *F*-tests reported have (1, 15) degrees of freedom.

One-way tests were utilised for all analysed predictions. It should also be noted that, as this paper details results for a number of dependent measures, the frequency of Type I errors increases. However, this increase in frequency of Type I errors cannot be controlled by adjusting α-levels, because the probability of Type I error remains the same [45].

## Results

**Fig. 1:**
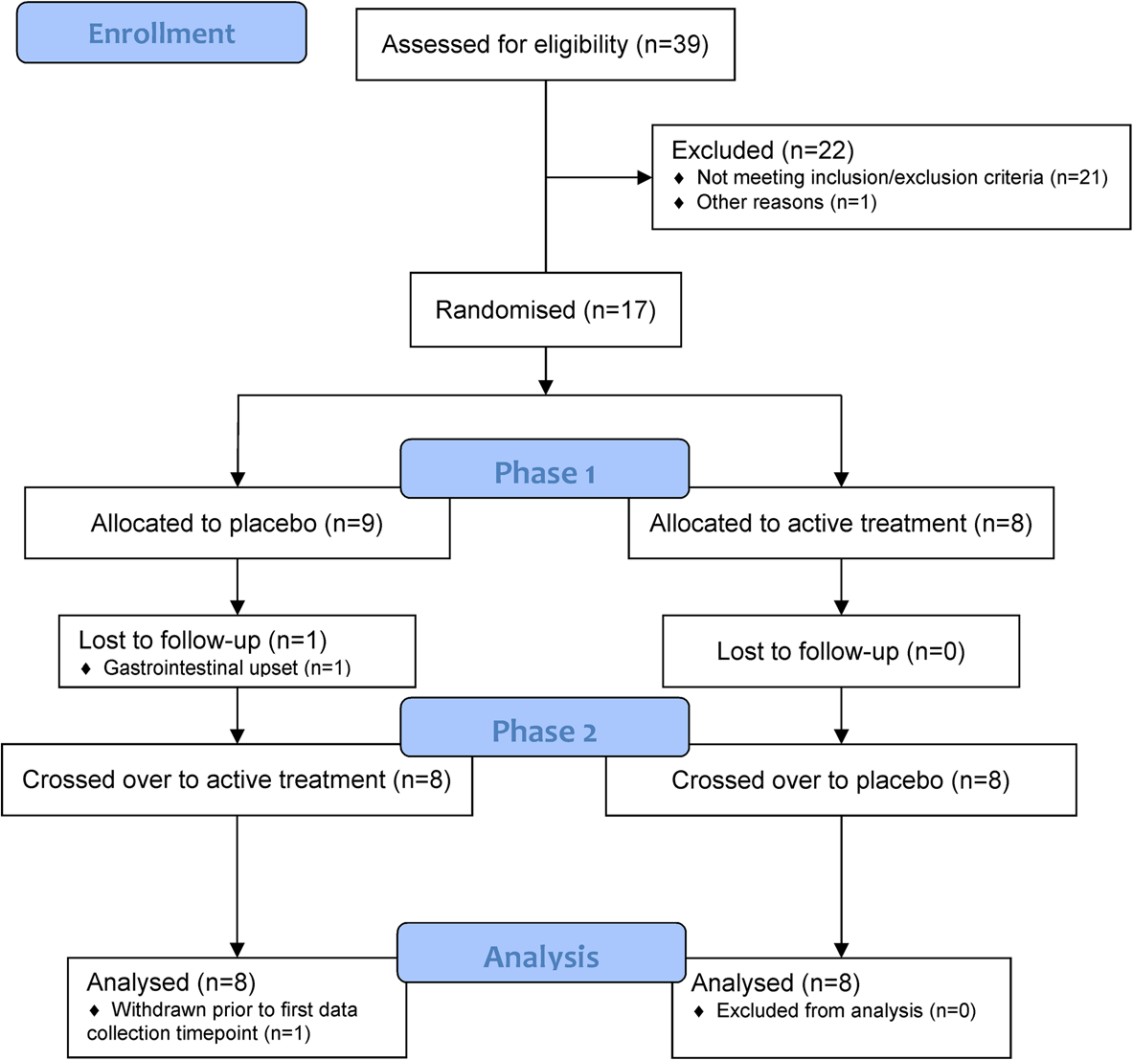
Flow chart of the study participants

As shown in Fig. 1, a total of 39 individuals expressed interest in the trial with 17 individuals randomised and 22 excluded due to failure to meet inclusion/exclusion criteria or availability constraints. Demographics and clinical characteristics of the 16 participants that completed the trial are presented in Table 1.

**Table 1:** Baseline demographics and clinical characteristics of participants

Demographics	Mean ± Standard Deviation
Age (years)	49.19 ± 14.28
Height (cm)	169.29 ± 9.35
Weight (kg)	70.50 ± 18.56
MMSE	29.50 ± 0.60
Pulse (bpm)	68.82 ± 14.27
Blood Pressure Systolic (mmHg)	118.71 ± 19.02
Blood Pressure Diastolic (mmHg)	76.29 ± 9.80

### Cognitive function

Table 2 displays the mean and standard error across subjects before and after treatment with SLT and placebo for each of the Compass tasks, together with measures of fatigue and alertness. Two of the Compass tasks showed effects of SLT that approached statistical significance. For the Alphabetic Working Memory Task, a decrease in reaction time from baseline to post-treatment was larger for SLT than placebo (*F* = 3.72, *p* = .073, *d* = 1.00). SLT also enhanced N-Back task performance, with greater accuracy reported after treatment (*cf.* baseline) with SLT than placebo (*F* = 4.35, *p* = .054, *d* = 1.09). The other Compass tasks and VAS mood and alertness scales did not show an effect of SLT (no treatment × time interaction).

**Table 2:** Mean difference and standard error between the SLT and placebo treatments for each Compass task

Compass Task	Mean Difference ± Standard Error
Immediate Word Recall (number correct)	0.19 ± 0.04
Delayed Word Recall (number correct)	0.69 ± -0.03
Word Recognition (number correct)	-0.06 ± 0.24
Picture Recognition (number correct)	-0.5 ± 0.23
Face Recognition (number correct)	0.31 ± -0.11
Simple Reaction Time (ms)	6.07 ± 7.54
2 Choice Reaction Time (accuracy %)	-0.88 ± 0.59
2 Choice Reaction Time (ms)	21.36 ± 11.82
4 Choice Reaction Time (accuracy %)	0.26 ± -0.18
4 Choice Reaction Time (ms)	-18.68 ± 0.68
Numerical Working Memory (accuracy %)	3.34 ± -1.12
Numerical Working Memory (ms)	90.09 ± 60.33
Alphabetic Working Memory (accuracy %)	0.13 ± -1.09
**Alphabetic Working Memory (ms)**	-**44.14 ±** -**19.78**
Corsi Block Span (Span Length)	0.25 ± -0.01
**N-Back (accuracy %)**	**4.17 ±** -**0.82**
Serial Subtract 3 (number correct)	1.63 ± -0.82
Serial Subtract 7 (number correct)	-0.94 ± -0.27
Rapid Visual Information Processing (accuracy %)	-2.16 ± 0.13
Rapid Visual Information Processing (ms)	40.67 ± -16.79
Mental Fatigue (End - Start)	-0.63 ± -2.32
Alertness (End - Start)	1.89 ± 0.40

Note: Tasks that are bolded had a treatment × time interaction that approached statistical significance (*p* < .10)

### ERP components

**Fig. 2:**
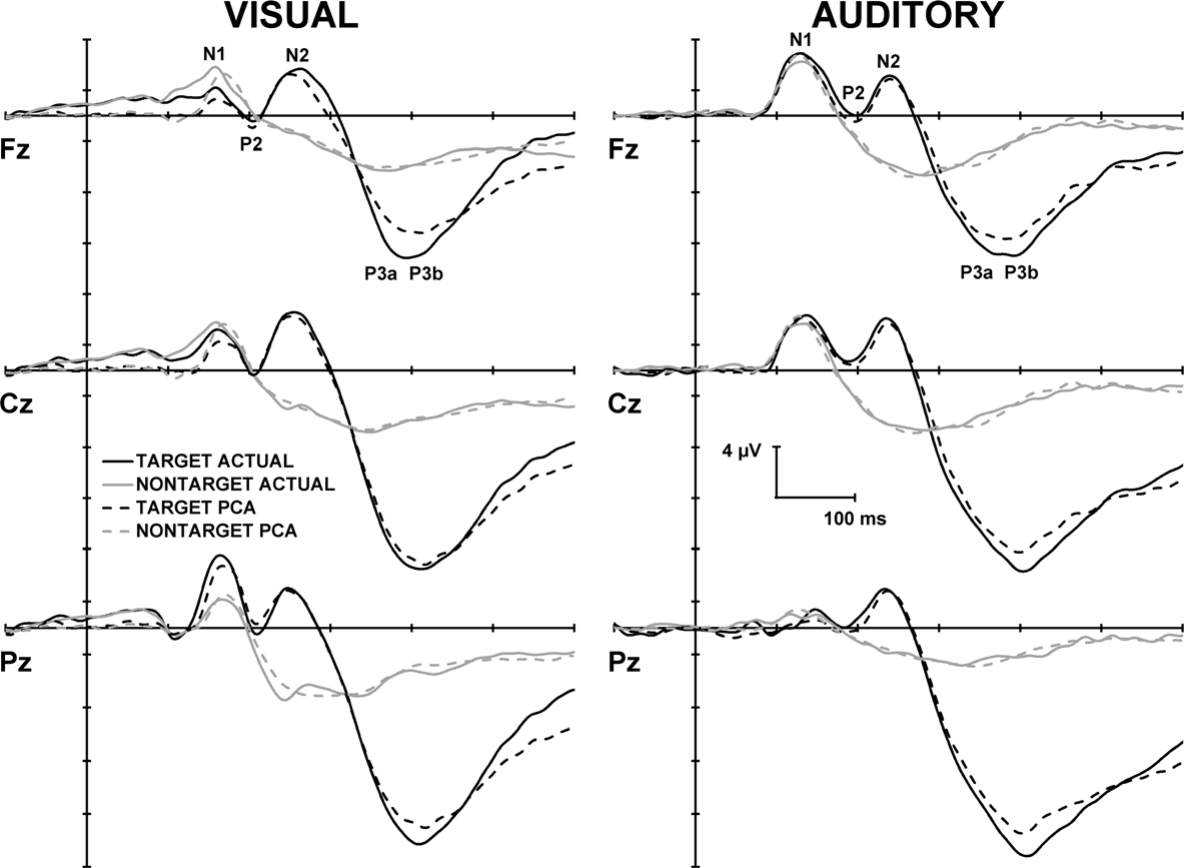
Grand mean ERPs for targets and nontargets from the midline sites for both visual (left) and auditory (right) modalities. The solid lines represent the raw data, and the dashed lines illustrate the reconstructed PCA waveforms. PCA extracted components are labelled at Fz for both modalities

Figure 2 illustrates the grand mean ERPs for targets and nontargets (solid lines) from midline sites (left: visual; right: auditory).

For the visual task PCA, factors 1–4 and factor 6 accounted for 84.9 % of the total variance, and for the auditory task PCA, the first 5 accounted for 87.8 % of the variance; these factors were extracted and retained for analysis. The sum of these extracted factors is illustrated in Fig. 2 (dashed lines) for each modality. When correlated across the midline, the reconstructed and original waveforms were highly similar for visual targets, *r*(1048) = .97, *p* = < .001, and nontargets, *r*(1048) = .92, *p* = < .001, and for auditory targets, *r*(1048) = .99, *p* = < .001, and nontargets, *r*(1048) = .98, *p* = < .001.

**Fig. 3:**
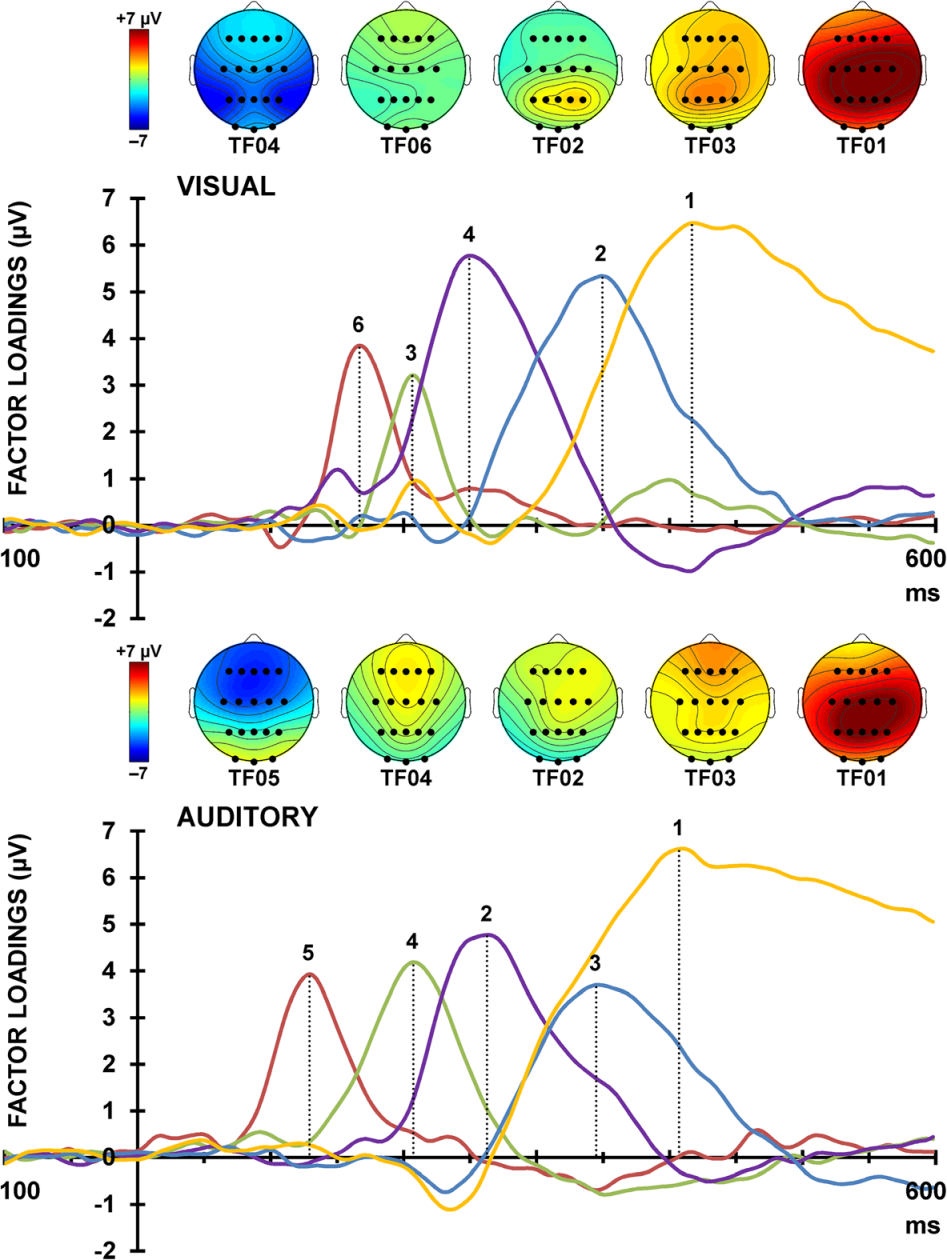
Factor loadings for visual (top) and auditory (bottom) modalities. The topographic headmaps for each of the extracted ERP components are shown above the factor loadings (averaged across all subjects, treatments, and testing sessions), separately for each modality

For each modality, the temporal factor loadings (rescaled to μV by multiplying each time point by the standard deviation [46]) for each of the ERP components are displayed as a function of time in Fig. 3. Topographic headmaps of the temporal components, averaged across treatment, time, and stimulus condition are displayed above the factor loadings for each of the modalities. Component labels, site of maximal amplitude, percentage of variance explained, and sites selected for analysis for each of the rotated components are indicated in Table 3. Components were labelled according to their polarity, latency, temporal sequence, comparison with the raw ERPs (see Fig. 2), and topography.

**Table 3:** Information for the factors identifiable as ERP components

Visual	TF004	TF006	TF002	TF003	TF001
Component	N1	P2	N2	P3a	P3b
Latency (ms)	168	208	252	352	420
Maximal Site	P3	O2	P4	P1	C2
Variance (%)	3.8	2.5	16.6	16.3	45.8
Sites Analysed	P3, P4	Fz	C3, Cz, C4	Fz, Cz, P3, Pz, P4	Cz, Pz
Auditory	TF005	TF004	TF002	TF003	TF001
Component	N1	P2	N2	P3a	P3b
Latency (ms)	132	210	264	348	412
Maximal Site	F2	Cz	Fz	Fz	Pz
Variance (%)	4.7	6.6	10.9	8.3	57.4
Sites Analysed	F3, Fz, F4, Cz	Fz, Cz	F3, Fz, F4, C3, Cz, C4	F3, Fz, F4	P3, Pz, P4

Note: Rows indicate component name, latency, site of maximal amplitude, percentage of total variance explained, and sites selected for analysis. TF = Temporal Factor

Table 4 details the mean and standard error across subjects for each analysed visual (middle) and auditory (lower) ERP component (targets and nontargets), at baseline and post-treatment, for both SLT and placebo. As shown in Table 4, amplitudes were significantly greater for targets than nontargets for visual N1 (*F* = 20.70, *p* < .001, *d* = 2.35), N2 (*F* = 20.60, *p* < .001, *d* = 2.35), and P3b (*F* = 34.26, *p* < .001, *d* = 3.06). There were no effects or interactions involving treatment and time for any of the visual ERP components.

**Table 4:** Mean difference and standard error between the SLT and placebo treatments for each EEG band and ERP component (all μV)

	Mean Difference ± Standard Error	
EEG Spectral Band	Eyes Open	Eyes Closed
Delta	-1.64 ± -0.01	-0.07 ± -0.73
Theta	-0.82 ± -0.37	0.20 ± -0.32
Alpha	-0.16 ± 0.44	-3.66 ± -0.94
Beta	2.14 ± 0.82	-0.65 ± -0.5
Visual ERP Component	Target	Nontarget
N1	-1.72 ± -0.06	-0.45 ± -0.17
P2	-0.37 ± -0.08	-0.69 ± 0.02
N2	-0.74 ± -0.06	-0.05 ± -0.26
P3a	2.58 ± 0.02	1.98 ± -0.20
P3b	-1.12 ± 0.10	-1.38 ± -0.04
Auditory ERP Component	Target	Nontarget
**N1**	**2.14 ± 0.11**	-**1.38 ±** -**0.14**
P2	1.16 ± 0.84	-1.02 ± -0.31
N2	-0.14 ± 1.78	-0.04 ± -0.13
**P3a**	**3.65 ± 0.07**	-**0.36 ±** -**0.08**
P3b	0.58 ± -0.25	-2.27 ± 0.10

Note: The bolded ERP components had a treatment × time × stimulus type interaction (*p* < .10)

**Fig. 4:**
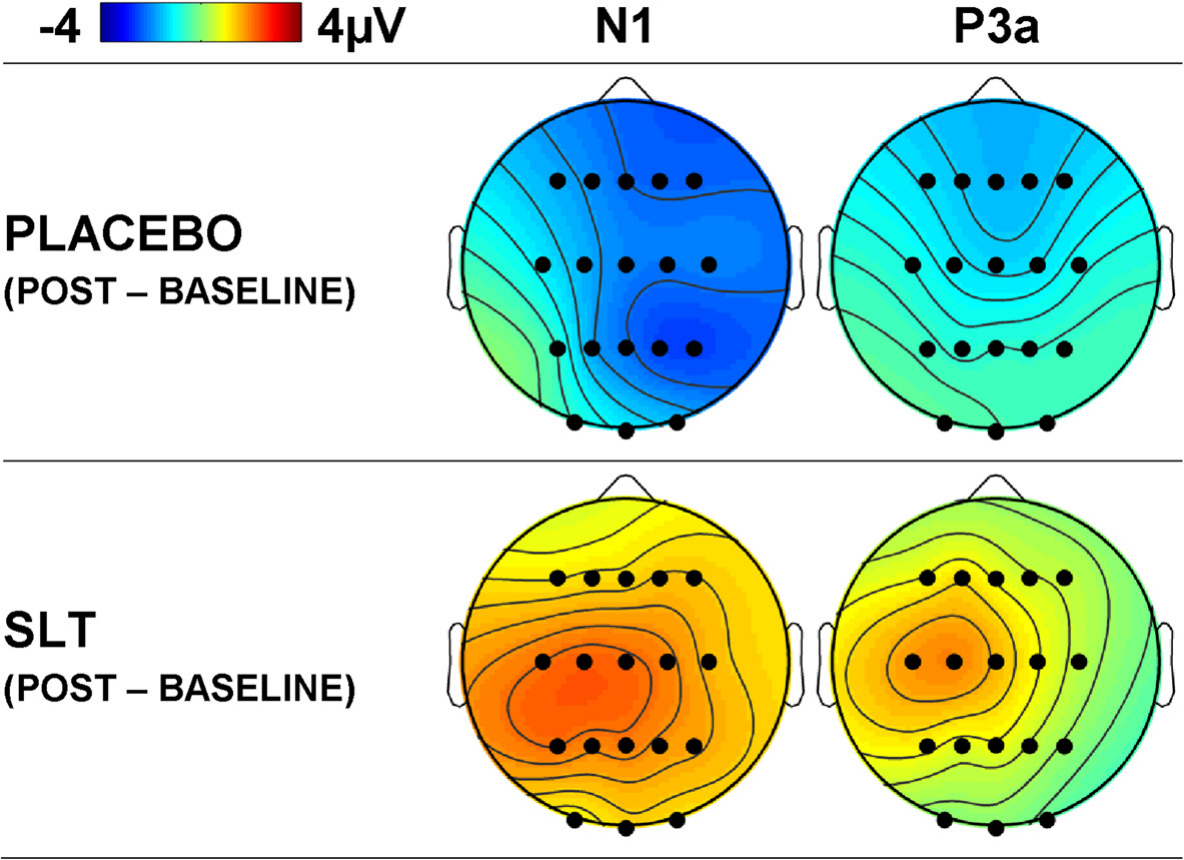
Mean difference topographic headmaps for auditory N1 and P3a. Headmaps illustrate the difference in stimulus condition (target minus nontarget) and the difference between sessions (post-treatment minus baseline) for placebo and SLT. A SLT related reduction in N1 negativity, and an increase in P3a positivity are apparent

Table 4 also illustrates significantly larger amplitudes to targets than nontargets for auditory N2 (*F* = 17.14, *p* = .001, *d* = 2.12), P3a (*F* = 10.61, *p* = .005, *d* = 1.67), and P3b (*F* = 88.73, *p* < .001, *d* = 4.96). As shown in Fig. 4, auditory target N1 was smaller following SLT compared to placebo (from baseline to post-treatment) than nontarget N1 (*F* = 6.39, *p* = .023, *d* = 1.31). For auditory P3a, the target enhancement showed a greater increase after treatment (*cf.* baseline) with SLT in comparison to placebo that approached significance (*F* = 3.15, *p* = .096, *d* = 0.91; see Fig. 4). Topographic headmaps (Fig. 4) illustrate that these differences were largest centrally and on the left. There were no other treatment × time interactions for the other auditory ERP components.

### EEG spectral bands

Delta and theta had a strong midline topography (defined as mean across Fz, Cz, Pz), and alpha and beta were both largest at parietal sites (mean across P3, Pz, P4). The across-subjects means and standard errors for each of the EEG bands, before and after both treatments, are detailed in Table 4, separately for eyes open and eyes closed conditions. Table 4 shows that amplitudes were greater for eyes closed than eyes open for both theta (*F* = 17.92, *p* = .001, *d* = 2.17) and alpha (*F* = 5.43, *p* = .034, *d* = 1.22) bands. There were no significant main effects or interactions for delta or beta. Treatment with SLT did not affect resting EEG amplitude for any of the four bands.

### Cardiovascular measures

Mean peripheral and aortic pulse pressure (mmHg; systolic minus diastolic difference), and HR (bpm) for placebo and SLT at baseline and post-treatment are detailed in Table 5. Treatment with SLT compared to placebo did not affect any of the three cardiovascular measures.

**Table 5:** Mean difference and standard error between the SLT and placebo treatments for each of the three cardiovascular measures

	Mean Difference ± Standard Error
Peripheral Pulse Pressure (mmHg)	-1.20 ± 0.52
Aortic Pressure (mmHg)	-2.50 ± 0.19
Resting HR (bpm)	1.07 ± 1.55

### Adverse effects

No serious adverse events were noted in this study. Minor adverse events outlined in Table 6 were noted during each cycle of the trial. One participant withdrew from the trial during the first intervention-cycle due to mild gastrointestinal symptoms which coincided with a probably unrelated upper respiratory infection. Symptoms were rated as moderate severity, onset was noted after two doses, and duration was three to four days. The participant was found to be treated with the placebo suggesting that these effects were unrelated to the trial.

**Table 6:** Frequency of adverse events during treatment cycles and washout period

	During Active Cycle	During Placebo Cycle	During Washout
Tiredness	3	2	3
Headaches	2	1	1
Loose Stools	1	1	0
Stomach Pain	1	0	0
Back Pain	1	0	0
Poor Sleep	0	1	0

## Discussion

This pilot study investigated the effect of SLT on neurocognitive and cardiovascular functioning in healthy adults. Participants were assessed before and after one week of treatment with 2 capsules of SLT (60 mg standardised extract per capsule) and placebo per day, separated by a one week washout period. Primary outcome measures were scores on a computerised neuropsychological test battery, and oddball task ERP component amplitudes. Secondary outcome measures were resting EEG band amplitudes and cardiovascular measures of central and peripheral pulse amplitude, and resting HR. There was a trend towards improvements in alphabetic working memory and visual working memory with SLT compared to placebo. Auditory P3a amplitudes were slightly larger following SLT than placebo for targets compared to nontargets, and a similar target enhancement showed a decrease following SLT for auditory N1. SLT did not significantly affect resting state EEG activity or cardiovascular system function.

On the Compass neuropsychological test battery, reaction time on the Alphabetic Working Memory Task and accuracy on the N-Back task both showed modest improvements following treatment with SLT, compared to placebo, suggesting that SLT may have the potential to improve working memory performance in healthy adults. Previous work has indicated that two of the constituents of SLT, *Ginkgo biloba* and *Panax ginseng*, have a similar effect on memory, with a large study (*N* = 256) showing significant improvements in numerical and spatial working memory after a 12 week trial of a *ginkgo*/*ginseng* combination [47]. A similar 30 day trial on adults [48] demonstrated significant improvements in working memory (along with processing speed and other aspects of executive function) following treatment with 120 mg *Ginkgo biloba* in comparison to placebo. Compared to the current pilot study, these previous studies used larger samples (*N* = 16 vs. *N* = 61–256) and different treatment doses (120 mg vs. up to 320 mg) for longer periods (1 week vs. 4–12 weeks), suggesting that the effect of SLT could be much greater given an increase in these parameters. In the current study, no effect of SLT was found on other Compass measures including Immediate and Delayed Word Recall, Word Recognition, Simple and Choice Reaction Time, Numeric Working Memory, Corsi Block Span, Picture Recognition, Face Recognition, Serial Subtraction, and the RVIP task, or on the mood and alertness scales.

From the visual and auditory oddball tasks, five ERP components were identifiable from each of the unrestricted temporal PCAs: N1, P2, N2, P3a, and P3b. Visual N1, N2, and P3b, and auditory N2, P3a, and P3b were all larger to targets than nontargets. These findings are broadly similar with previous oddball studies [49–54]. As the purpose of this study was to evaluate the effects of SLT, these target/nontarget differences together with the components that were not sensitive to treatment with SLT (visual N1, P2, N2, P3a, and P3b, and auditory P2, N2, and P3b) will not be discussed further.

Target N1 was smaller following SLT compared to placebo (from baseline to post-treatment) than nontarget N1. This is a novel finding that has not been reported elsewhere in the literature. To the best of our knowledge, only one other study has examined changes in N1 amplitude after treatment with any SLT constituents. In that study [53], no changes in auditory oddball N1, P2, N2, or P300 amplitudes were reported after acute and chronic doses of *Ginkgo biloba.* The N1 component elicited in the current study had a strong fronto-central topography and latency (132 ms) suggestive of N1 Component 1 [54]. This component is generated in the supratemporal plane of the primary auditory cortex [55], and it is thought to facilitate the conscious perception of auditory stimuli [50]. A reduction in amplitude indicates less activation in these regions. Although it is difficult to speculate about any functional significance of this treatment-related change, findings might reflect more efficient attentional processing of auditory information.

Auditory P3a amplitudes trended towards being larger following SLT than placebo for targets compared to nontargets; another novel finding. P3a, is elicited to attention-capturing stimuli [56, 57] and is thought to relate to working memory processes [58], with evidence from lesion studies suggesting that its neural generators are located in the frontal lobe, hippocampus, and thalamus [59; for a review see 60]. Speculatively, and when viewed in conjunction with the Compass test findings, this SLT-related enhancement in P3a amplitude, may represent increased working memory activation. It should also be noted that an analysis of P3a has not been reported in similar herbal medicine work as most studies quantify component amplitudes using baseline-to-peak measures. It is important to investigate overlapping components using techniques such as PCA (as done in the current study) in order to fully understand the (often subtle) effects of various interventions. There were no SLT treatment-related effects on the other visual and auditory ERP component amplitudes.

The topographic distribution for each of the EEG bands was in line with previous work examining resting condition amplitudes [61]. Delta and theta were largest at the midline, and alpha and beta were both maximal parietally. Typical condition-related changes in amplitudes were apparent with larger amplitudes for eyes closed than eyes open for theta and alpha bands; a pattern of results broadly in line with other work [62–63]. Treatment with SLT did not have an effect on resting EEG activity.

Mean aortic and peripheral pulse pressure, and resting HR did not show any changes with SLT treatment. This is not surprising given that the current study recruited healthy adults, and special care was taken to ensure that older participants, who are at increased risk of cardiovascular disease, had a full blood count that was within normal limits.

### Limitations and future directions

This was the first study to assess SLT, a formula with a unique combination of three herbs, in a healthy cohort. Compared to this study, previous work [47, 48] using *Ginkgo biloba* and/or *Panax ginseng* found significant improvements in a range of executive functions, such as episodic memory, in addition to improved mood and arousal (for a review see [64]). Those studies used larger sample sizes (*N* = 61–256), longer interventions (4–12 weeks), and larger treatment doses (up to 320 mg per day) than the current study. Given the substantial preclinical work showing the potential benefits of SLT, and the increase in therapeutic effects due to synergism, further research in a larger cohort is needed, with increases in the parameters mentioned above.

Future work is also required to replicate the novel N1 and P3a amplitude findings, and should also include latency analyses. Previous research [52, 65] has reported changes in latency, rather than amplitude, however, latency analyses are not possible with temporal PCA. Future work could complement PCA analyses with Residue Iteration Decomposition (RIDE) analysis, which uses latency variability to estimate single trial latencies with a high degree of precision [66–68]. A study combining these analyses would be able to examine any treatment-related changes in ERP components effectively.

As with the neurocognitive measures, the dose and treatment length of SLT were probably too small to affect cardiovascular parameters in healthy adults. Further, there is some evidence suggesting that acute, rather than chronic, administration of *ginkgo* may produce larger effects. For instance, another study employed radial pulse wave analysis to measure reflection, stiffness, and peripheral augmentation indices and reported improvements in the stiffness index 2 h after treatment [69]. Future work could examine both the acute and chronic effects of SLT on cardiovascular function in healthy adults.

## Conclusions

Although the observed effects of SLT were small, findings indicate that SLT has the potential to improve working memory performance in healthy adults. A larger sample size, and possibly a higher dose of SLT over a longer period are needed to extend upon these results. A dose escalation study to determine the appropriate dosage for cognitive benefits is recommended. The possible benefits of SLT should also be explored in healthy older adults, and individuals with mild cognitive impairment.

## Abbreviations

SLT: Sailuotong
EEG: Electroencephalograph
ERP: Event-related potential
CHM: Chinese Herbal Medicine
AChE: Acetylcholinesterase
NMDA: N-methyl-D-asparate
ADAS-cog: Alzheimer’s Disease Assessment Scale cognitive subscale
SPECT: Single Photon Emission Computed Tomography
HR: Heartrate
RVIP: Rapid Visual Information Processing
VAS: Visual Analogue Scale
RT: Response Time
PCA: Principal Components Analysis
